# Repeated evolution of soldier sub-castes suggests parasitism drives social complexity in stingless bees

**DOI:** 10.1038/s41467-016-0012-y

**Published:** 2017-02-23

**Authors:** Christoph Grüter, Francisca H. I. D. Segers, Cristiano Menezes, Ayrton Vollet-Neto, Tiago Falcón, Lucas von Zuben, Márcia M. G. Bitondi, Fabio S. Nascimento, Eduardo A. B. Almeida

**Affiliations:** 1 Departamento de Biologia, Faculdade de Filosofia, Ciências e Letras de Ribeirão Preto, Universidade de São Paulo, CEP 14040-901, Ribeirão Preto, São Paulo Brazil; 2 Embrapa Amazônia Oriental, Belém, CEP: 66095-903 Pará Brazil; 3 Departamento de Genética, Faculdade de Medicina de Ribeirão Preto, Universidade de São Paulo, CEP: 14049-900 Ribeirão Preto, São Paulo Brazil; 4 Institute of Zoology, Johannes Gutenberg University Mainz, Johannes von Müller Weg 6, 55099 Mainz, Germany

## Abstract

The differentiation of workers into morphological castes represents an important evolutionary innovation that is thought to improve division of labor in insect societies. Given the potential benefits of task-related worker differentiation, it is puzzling that physical worker castes, such as soldiers, are extremely rare in social bees and absent in wasps. Following the recent discovery of soldiers in a stingless bee, we studied the occurrence of worker differentiation in 28 stingless bee species from Brazil and found that several species have specialized soldiers for colony defence. Our results reveal that worker differentiation evolved repeatedly during the last ~ 25 million years and coincided with the emergence of parasitic robber bees, a major threat to many stingless bee species. Furthermore, our data suggest that these robbers are a driving force behind the evolution of worker differentiation as targets of robber bees are four times more likely to have nest guards of increased size than non-targets. These findings reveal unexpected diversity in the social organization of stingless bees.

## Abstract (web summary)

Although common in ants and termites, worker differentiation into physical castes is rare in social bees and unknown in wasps. Here, Grüter and colleagues find a guard caste in ten species of stingless bees and show that the evolution of the guard caste is associated with parasitization by robber bees.

## Introduction

Division of labor among cells, organs, or individuals is a fundamental feature of complex biological systems^1–3^. In social insects, division of labor among workers is widespread and the most advanced forms of division of labor are found in species with morphologically distinct worker phenotypes^4–6^. In many ant and termite species, for example, colony defence is performed by a soldier caste (or sub-caste)^4–6^. Having workers with morphological adaptations for specific tasks such as foraging or defence is likely to improve colony functioning and performance because workers are more efficient at performing these tasks^7–9^. For example, more specialized ant soldiers (or majors) are more effective at nest defense^10^, whereas minors are better at brood care^11^. Given the benefits of task-related worker differentiation, it is puzzling that physical worker castes are extremely rare in social bees^9^ and absent in wasps^6^. It has been argued that developmental constraints^12,13^, individual-level selection^5,13^, the presence of a powerful sting^5^ or the fact that colonies with winged workers can more easily avoid aggressive interactions^5^ might prevent the evolution of physical castes in bees and wasps.

Division of labor is mainly based on temporal castes in both groups of highly eusocial bees, the honey bees (Apini) and the stingless bees (Meliponini)^5,6,14,15^: workers first perform nursing duties inside the nest before moving on to general nest maintenance duties and, finally, they perform the outside tasks of guarding and foraging. However, the recent discovery of soldiers in the Neotropical stingless bee *Tetragonisca angustula* ^9^ suggests that more complex caste systems might exist in this relatively understudied tribe. In *T. angustula*, colonies are defended by a small^16^ but dedicated group^17^ of entrance guards that are both larger (~ 30%) and of different shape than their nestmates^9^. Having larger soldiers is beneficial for colonies because body size is directly linked to the fighting ability of *T. angustula* guards^9^.

Given this discovery in a common Neotropical species, we tested if task-related worker differentiation is more widespread in stingless bees, the largest group of eusocial bees (>500 described species^18^). To this end, we compared the morphology of nest guards and foragers of 28 species from different areas in Brazil. We chose species that are both relatively common and ecologically varied: they show diversity in their habitat (e.g., savanna, subtropical forests, and tropical rain forest) (see Supplementary Table 1), nesting habits (ground nesting, cavity nesting, and exposed nests)^19^, foraging method (e.g., pollen foraging, necrophagous, and cleptoparasitic)^20^ and colony size (from a few hundred to tens of thousands of workers)^21,22^. We focused on nest entrance guards and foragers because worker differentiation in ants and termites (and the stingless bee *T. angustula*) mostly involves morphological adaptations for defence and foraging^5,6,9,23^. Furthermore, we scrutinized existing hypotheses that might explain the evolution of worker sub-castes. Our results show that worker differentiation is indeed common in Neotropical stingless bees and that the evolution of nest-entrance guards of increased body size is linked to the risk of being attacked by parasitic robber bees.

## Results

### Differences between guards and foragers

**Fig. 1:**
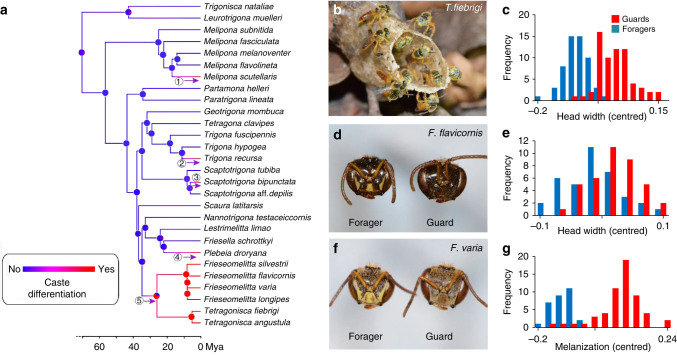
**Comparison of guards and foragers in 28 species of stingless bees.** **a** Phylogenetic reconstruction based on a previously published phylogeny^18^. The color gradient from *blue* to *red* indicates the probability that a species evolved increased guard size, based on 1,000 simulations using a Bayesian framework. Numbers 1–5 indicate independent appearances of increased guard size. **b** *Tetragonisca fiebrigi* guards standing on the wax entrance tube (Photo: C. Grüter). **c** Size-frequency distribution of *T. fiebrigi* foragers and guards showing a bimodal distribution. Values (unit = mm) are centred for each colony (colony mean and total mean = 0) to correct for overall colony differences (*N* = 58 forager/65 guards/6 colonies). **d** *Frieseomelitta flavicornis* guard and forager (Photo: C. Grüter). **e** Size-frequency distribution of *F. flavicornis* foragers and guards, showing a unimodal distribution. Values (unit = mm) are centred for each colony (*N* = 37/39/6). **f** Head of a *Frieseomelitta varia* forager and guard (Photo: C. Grüter). **g** Melanization frequency distribution of *F. varia* guards and foragers. Values (unit = melanization level, see methods) are centred for each colony (*N* = 30/56/6)

We found that guards were significantly larger than foragers in 10 out of 28 species (in 6 of 16 genera) (Fig. 1a; Table 1 and Supplementary Table 1). The species with larger guards had an overall greater worker size variation (phylogenetically controlled generalized least squares (GLS): *t*-value = 2.27, df = 26, *P* = 0.03). In several species, the size difference between guards and foragers was larger than one standard deviation of within colony worker size variation (i.e., differentiation index DI > 1, Table 1, Supplementary Fig. 1). The three species with the largest degree of size differentiation, *T. angustula* (DI = 1.6), *Tetragonisca fiebrigi* (DI = 1.54) and *Frieseomelitta longipes* (DI = 1.35) show a bimodal size distribution (Fig. 1b, c; Supplementary Fig. 1). In the other seven species with worker size differentiation, guard, and forager sizes show considerable overlap (Fig. 1d, e; Supplementary Fig. 1).

**Table 1:** Summary of morphological data

**Species**	Head width (mm)	Size difference_G-F_	CV_Headwidth_	DI	Guards vs. Foragers		Allometry vs. Isometry			Weight (mg)	CV_Weight_	Target of ***Lestrimelitta*** ^a^
					***t***-value	***P***-value*	Slope	***t***-value	***P***-value*			
*Friesella schrottkyi*	1.41 ± 0.033	−0.5%	0.0239	0.23	−0.99	0.51	0.69	1.83	0.1	2.81 ± 0.28	0.1033	Yes
*Frieseomelitta flavicornis*	2.26 ± 0.045	1.6%	0.0203	0.88	3.55	***0.0051***	0.37	4.1	***0.0012***	9.49 ± 1.5	0.1646	No
*Frieseomelitta longipes*	2.37 ± 0.029	1.7%	0.0129	1.35	2.96	***0.025***	0.41	2.16	0.073	10.8 ± 1.7	0.1594	No
*Frieseomelitta silvestrii*	1.74 ± 0.033	1.3%	0.0195	0.67	2.59	***0.039***	0.72	1.17	0.26	5.05 ± 0.44	0.0897	Yes
*Frieseomelitta varia*	2.33 ± 0.036	1.1%	0.0156	0.74	3.38	***0.0051***						Yes
*Geotrigona mombuca*	2.45 ± 0.035	0.9%	0.0142	0.63	2.12	0.082	0.55	1.76	0.1	12.37 ± 1.4	0.1184	No
*Lestrimelitta limao*	2.19 ± 0.032	0.2%	0.0144	0.17	0.67	0.58	0.61	2	0.078	12.11 ± 0.86	0.0713	No
*Leurotrigona muelleri*	1.11 ± 0.024	1.2%	0.0217	0.58	2.09	0.082	0.51	2.55	***0.039***	1.4 ± 0.24	0.1705	No
*Melipona fasciculata*	4.45 ± 0.048	0.2%	0.0113	0.16	0.64	0.58	0.55	2.27	0.062	101.6 ± 7.2	0.0721	No
*Melipona flavolineata*	3.77 ± 0.075	0.6%	0.0202	0.31	1.08	0.5	0.55	2.14	0.073	56.4 ± 5.9	0.1063	No
*Melipona melanoventer*	4.4 ± 0.063	−0.1%	0.0119	0.10	−0.4	0.69	0.4	3.92	***0.0016***	92.2 ± 11.0	0.1187	No
*Melipona scutellaris*	4.06 ± 0.071	1.8%	0.0178	1.05	3.38	***0.0076***	0.67	1.75	0.1	73.5 ± 6.2	0.0872	Yes
*Melipona subnitida*	3.7 ± 0.052	0.9%	0.0141	0.64	2.28	0.064	0.4	2.77	***0.033***	60.4 ± 7.2	0.1217	No
*Nannotrigona testaceicornis*	1.88 ± 0.03	−0.5%	0.0160	0.34	−1.33	0.34	0.71	2.17	0.07	6.74 ± 0.53	0.0804	Yes
*Paratrigona lineata*	1.73 ± 0.029	0.4%	0.0169	0.24	0.74	0.58	0.33	3.77	***0.003***	6.27 ± 0.91	0.1469	No
*Partamona helleri*	2.52 ± 0.031	0.2%	0.0121	0.15	0.59	0.6	0.74	1.38	0.19	15.97 ± 0.90	0.0565	No
*Plebeia droryana*	1.63 ± 0.036	1.1%	0.0221	0.50	2.46	***0.045***	0.69	2.05	0.073	4.24 ± 0.43	0.1035	Yes
*Scaptotrigona bipunctata*	2.74 ± 0.058	1.8%	0.0210	0.99	4.51	***0.0051***	0.53	2.66	***0.033***	19.01 ± 2.11	0.1107	Yes
*Scaptotrigona* aff. *depilis*	2.65 ± 0.034	1.3%	0.0129	1.00	2.31	0.06	0.82	0.97	0.34	16.49 ± 0.99	0.0614	No
*Scaptotrigona tubiba*	2.28 ± 0.035	0.3%	0.0152	0.17	0.7	0.58	—	—	—	10.89 ± 1.28	0.1192	No
*Scaura latitarsis*	1.73 ± 0.03	−0.3%	0.0177	0.17	−0.64	0.58	—	—	—	—	—	Yes
*Tetragona clavipes*	2.51 ± 0.035	−0.3%	0.0141	0.23	−0.91	0.55	0.67	1.95	0.083	12.2 ± 1.03	0.0854	No
*Tetragonisca angustula*	1.79 ± 0.066	5.9%	0.0380	1.59	11.75	***<0.0001***	0.81	2.48	***0.04***	4.62 ± 0.67	0.1481	Yes
*Tetragonisca fiebrigi*	1.79 ± 0.056	4.8%	0.0315	1.54	12.45	***<0.0001***	—	—	—	—	—	Yes
*Trigona fuscipennis*	2.6 ± 0.032	0.2%	0.0124	0.14	0.65	0.58	0.57	2.65	***0.033***	15.51 ± 0.98	0.0634	No
*Trigona hypogea*	2.28 ± 0.025	0.3%	0.0109	0.23	1.01	0.51	0.38	4.09	***0.0012***	10.54 ± 0.85	0.0805	No
*Trigona recursa*	2.31 ± 0.055	1.9%	0.0242	0.80	3.52	***0.0051***	0.73	1.77	0.1	11.28 ± 1.19	0.1067	No
*Trigonisca nataliae*	1.17 ± 0.014	0.1%	0.0122	0.12	0.4	0.69	0.54	1.75	0.1	1.5 ± 0.12	0.0812	No

Mean head size (± s.d.) and head size differences between guards and foragers, differentiation index (DI), allometry between body weight and head size are shown for the studied species

*CV* coefficient of variation, *DI* differentiation index

*P-values shown are after Benjamini & Hochberg^46^ correction for false discovery rate. Significant *P*-values are bold and italic.

^a^Known targets according to a recent survey^29^

We also discovered that in several *Frieseomelitta* species*,* guards are not only larger but also constitute a distinct color morph (Fig. 1d, f, g; Supplementary Fig. 2a). The degree of melanization differed significantly between guards and foragers (*F. varia*, linear mixed-effects (LMEs), *t*-value = 13.45, df = 81, *P* < 0.001, DI = 1.7; *F. flavicornis*, *t*-value = 8.84, df = 71, *P* < 0.001, DI = 1.5; *F. longipes*, *t*-value = 11.5, df = 23, *P* < 0.001, DI = 1.85). We measured cuticle thickness (i.e., sclerotization) in the clypeal area using transmission electron microscopy but found no difference between guards and foragers (LME, *t*-value = 1.57, df = 6, *P* = 0.16).

We found negative allometry between body weight and head width in 9 of 24 tested species (Table 1). In other words, larger workers have relatively smaller heads. There was no association between negative allometry and having larger guards (Pagel’s method^24^ for correlated evolution: likelihood ratio = 0.06, *P* = 0.97) (Table 1), indicating that negative allometry in stingless bees is not linked to the morphological differentiation between defence workers and foragers.

### Testing hypotheses explaining worker differentiation

**Fig. 2:**
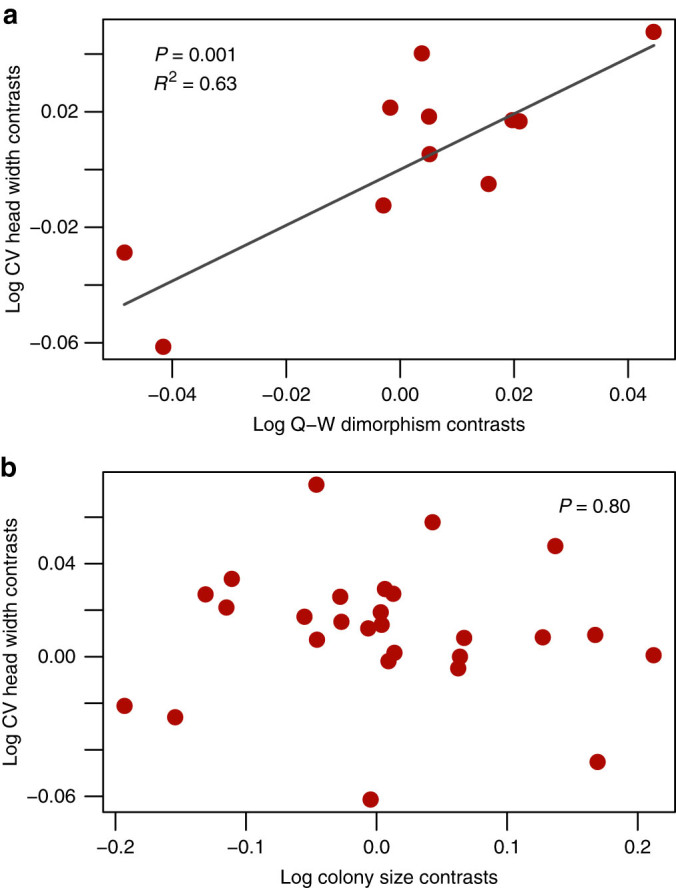
**Factors explaining differences between species in worker diversity.** **a** Relationship between queen-worker dimorphism (log-transformed) and worker diversity (log-transformed standard deviation of head width) (*N* = 12 species). The phylogeny is based on ref. 18 and a generalized least squares model (GLS) assuming Brownian motion was applied. The best fit line is based on a linear regression through the origin based on phylogenetically independent contrasts (PICs). Queen-worker dimorphism explained 63% of the variation in worker size variation between species. **b** Relationship between colony size (log-transformed) and worker diversity (log-transformed standard deviation of head width) (*N* = 27 species). A GLS model revealed no relationship between colony size and worker diversity

 Our data allowed us to examine hypotheses that might explain inter-specific variation in the degree of worker differentiation in stingless bees. The developmental constraints hypothesis predicts a positive correlation between the variance in worker size and queen-worker dimorphism^13^, because an early queen-worker caste determination (and, therefore, greater Q-W dimorphism) provides more time for worker larvae to develop along different developmental pathways^13,25^. We performed a phylogenetically controlled analysis and found strong support for this prediction (GLS; *t*-value = 4.47, df = 10, *P* = 0.0012; Fig. 2a). Queen-worker dimorphism (i.e*.,* relative size difference) explained more than 60% of the variation in worker diversity between species.

The size-complexity hypothesis predicts that species with larger colony sizes have a more specialized division of labor and a more diverse workforce^3,26–28^. However, we found no relationship between colony size and worker size variation in our 28 species of stingless bees (GLS: *t*-value = −0.25, df = 26, *P* = 0.80) (Fig. 2b). We then tested if species with a significant difference in forager and guard size have larger colonies than species without, but we found no difference in colony size (GLS: *t*-value = 0.11, df = 26, *P* = 0.92).

### Phylogenetic analysis

 A reconstruction of the evolutionary history of worker differentiation suggests that the common ancestor of the species included in our study had similarly sized guards and foragers (Fig. 1a). The analysis further suggests that increased guard size evolved five times independently among the 28 study species (Fig. 1a). All transitions towards increased guard size have occurred relatively recently, during the last 20–25 million years (Fig. 1a). This period coincides with the period of diversification of the cleptoparasitic genus *Lestrimelitta* from non-parasitic ancestors (Fig. 1a). According to a recent survey, 10 of the 28 studied species are known targets of *Lestrimelitta,* whose attacks frequently destroy colonies^29^. Targets of robber bees are about four times more likely to have larger guards (70% or 7 of 10 species) than non-target species (16.7% or 3 of 18). We again used Pagel’s method^24^ to test for a correlated evolution of binary characters and found that species are significantly more likely to have guards of increased size if they are victims of *Lestrimelitta* robber bees (likelihood ratio = 8.17, *P* = 0.017).

## Discussion

Our results show that task-related worker differentiation is relatively common in stingless bees, with 10 of the 28 tested species having entrance guards that are significantly larger than the foragers of the same colonies. The three species with the most pronounced size differentiation, *T. angustula*, *T. fiebrigi,* and *F. longipes* show a bimodal size distribution (Fig. 1b, c; Supplementary Fig. 1), which indicates that guards and foragers represent distinct minor and major (soldier) sub-castes^6,12^. In the other seven species with worker size differentiation, guard and forager sizes, although significantly different, show considerable overlap. This size overlap among worker groups performing different tasks is similar to some bumble bee species, where body size is linked to task performance, but workers performing different tasks come from the same size distribution^30^. These findings show that the extent of worker differentiation in stingless bees varies among species, from guards of slightly increased size to distinct soldiers.

We discovered that in several *Frieseomelitta* species*,* guards are not only larger but also constitute a distinct color morph (Fig. 1d, f, g; Supplementary Fig. 2a). Guards are significantly darker than foragers, indicating stronger sclerotization, but measurements of cuticle thickness in the clypeal area did not show differences between guards and foragers. It is possible that camouflage explains color differences as the darker color makes entrance guards less conspicuous when they defend the nest entrance (Supplementary Fig. 2b). Increased melanization can also increase resistance to pathogens and physical damage^31^, which might be particularly beneficial for individuals that are more likely to encounter these risks. In some stingless bees and the honey bee (*Apis mellifera*), guarding tasks precede foraging activities^14,15,17^ and it is possible that *Frieseomelitta* guards change melanization after some days to become yellow foragers. However, behavioral observations in *F. varia* showed that workers performing defensive tasks (guarding and fighting) were of similar age (40.0 ± 5.1 days) to foragers (37.2 ± 9.2 days)^32^. This mirrors the situation found in *T. angustula,* where soldiers and foragers are of the same age^17^ and suggests that guarding and foraging are not sequential tasks in *Frieseomelitta*, but that the larger, dark guards represent a distinct soldier caste.

Allometry between body parts means that workers of different sizes differ in shape^6,25^. We found negative allometry between body weight and head width in 9 of 24 tested species. In other words, larger workers have relatively smaller heads, which is the opposite of what is found in many ant species^6^. Importantly, there was no association between negative allometry and having larger guards, which suggests that negative allometry does not seem to be linked to the size differentiation between defence workers and foragers in stingless bees. Guards in stingless bees might not need large heads accommodating strong head muscles as, in contrast to ants, they mainly use their mandibles for clamping, rather than cutting, and their legs for grappling^9,33^. Why some stingless bees show negative allometry and whether developmental constraints or ecological pressures play a role is unknown.

Compared to the impressive size differences between soldiers and minors in some ant genera (e.g., *Atta),* size differences between guards and foragers in stingless bees are moderate where they exist. This raises the question why more extreme worker differences have not evolved in eusocial bees. Several non-mutually exclusive hypotheses have been proposed to explain differences among social insect species in the degree of worker differentiation^5,13^. The developmental constraints hypothesis predicts that worker phenotypic diversity is more likely to evolve in species with an early queen-worker caste determination as this provides more time for worker larvae to develop along different developmental pathways^13,25^. As a result, we expect a positive correlation between the variance in worker size and queen-worker dimorphism^13^. Indeed, we found that queen-worker dimorphism explained more than 60% of the variation in worker diversity between species. This positive correlation between queen-worker dimorphism and worker phenotypic diversity could also be caused by a third factor, namely the reproductive potential of workers. A decreasing ability of workers to reproduce could favor both the evolution of worker differentiation^13^ and queen-worker dimorphism^34^. However, Tóth et al.^21^ found no link between worker reproduction and queen-worker dimorphism in stingless bees. Thus, we deem it unlikely that worker reproductive potential explains the strong relationship between queen-worker size difference and worker diversity.

The size-complexity hypothesis predicts that species with larger colony sizes have a more specialized division of labor and a phenotypically more diverse workforce^3,26–28^. There is good evidence for a size-complexity relationship within ant species^6^, but evidence among ant species is mixed^13,27^. We found no relationship between colony size and worker size variation among our 28 species of stingless bees (Fig. 2b), which suggests that colony size does not explain the presence or absence of worker differentiation in stingless bees.

A reconstruction of the evolutionary history of worker differentiation suggests that the common ancestor of our 28 species had similarly sized guards and foragers and that, therefore, increased guard size is a derived trait (Fig. 1a). The analysis further suggests that increased guard size evolved five times independently among the 28 study species (Fig. 1a). Stingless bees began their diversification around 80 million years ago^18^, but all transitions towards increased guard size have occurred relatively recently, during the last 20–25 million years (Fig. 1a). This period coincides with the period of diversification of the cleptoparasitic genus *Lestrimelitta* from non-parasitic ancestors (Fig. 1a). *Lestrimelitta* bees pose a threat to many Neotropical species as robbers of resources^29^ and are suspected to be the driving force behind the evolution of soldiers in *T. angustula* ^9,16^. For example, *T. angustula* soldiers aggressively attack objects that chemically or visually resemble robber bees^35^ and colonies have more soldiers in areas where *Lestrimelitta* attacks are more common^16^. Ten of the 28 studied species are known victims of *Lestrimelitta,* whose attacks frequently destroy colonies^29^. We found that victims of robber bees are significantly more likely to have larger guards than non-target species (70% vs*.* 16.7%). Having larger guards could benefit colonies as body size is likely to affect the ability to fight off intruders^9^.

Four of five transitions towards increased guard size occurred during the last 10 million years, a time period of global cooling^36^ and an increase in seasonality^37^. It is possible that these climatic changes further amplified the need to store and defend energy sources, such as honey and pollen. Michener^38^, for example, observed that stingless bees store more honey in cooler climates. In honey bees, the need for colony defence dramatically increases as foraging conditions worsen towards winter and colonies start to rob honey from other colonies^39^. However, honey bees and eusocial wasps might not need specialist soldiers because all workers are equipped with a powerful sting^5^. In stingless bees, the emergence of dangerous robbers and an environment that favored the storage of valuable resources might represent two important incentives for having larger and, therefore, stronger entrance guards. Previous studies have shown that a more dangerous environment can lead to increased investment in colony defence in species that already have soldiers^16,40^. Our study extends this theme and identifies a specific threat (*Lestrimelitta* robber bees) as a possible selective force for the evolution of new sub-castes and increased social complexity in Neotropical stingless bees.

## Methods

### Study species

Colonies originated from five different regions in Brazil (Supplementary Table 1). We used both wild colonies and colonies kept in wooden hive boxes. All colonies foraged on natural food sources. We estimated relative colony size by measuring colony traffic at the entrance^16^. We counted bees entering a colony for 1–2 min on a day with good foraging conditions, once in the morning and once in the afternoon (between 10 a.m.–12 noon and 1 p.m.–3 p.m.). For analysis, we averaged the measurements per colony and the colony measurements per species to obtain a species specific estimate of relative colony size (Supplementary Table 1) (no traffic data was available for both time periods for *Scaura latitarsis* due to the remoteness of the sampling location). Our values correlate well with published colony size estimates (correlation-coefficient *r* = 0.88, *N* = 11 species; estimates taken from^21^).

### Capture of workers

We studied 3.8 ± 1.8 colonies (mean ± s.d., range: 2–8) and 70.5 ± 22.6 workers (range: 28–123) per species (Supplementary Table 1; collection licenses: Ibama#26649-5 and SISBIO 27254-1). For forager collection, we caught returning pollen foragers at the colony entrance in 26 species. In the cleptoparasitic *Lestrimelitta limao* and the necrophagous *Trigona hypogea*, two species that do not visit flowers, we collected bees that were leaving the nest. Nests of stingless bees are defended by entrance guards^19,22^. Guards were identified by their typical defensive posture at the entrance and then captured. We used forceps, plastic bags, or Eppendorf tubes to capture bees, depending on the species and worker type. Other methods, such as netting of several bees at the entrance have been used in other studies^41^, but they do not allow for a distinction between guards and foragers.

### Guard and forager size measurements

Bees were freeze-killed and their head width and wet weight (without pollen) measured. The head was removed from the body and laid onto a 1 mm graph paper. We photographed the heads with a Nikon D7000 digital camera with a macro lens (Nikon AF-S VR 105 mm), positioned at a standardized distance from the graph paper. Subsequently, head width was measured using ImageJ 1.46 (measurement error: ± 0.01 mm, *N* = 46 measurements). Weight was measured to the nearest 0.1 mg using a Sartorius TE64 high precision balance. In two species, wet weight was not measured because the remote sampling locations made this impossible. Additionally, workers of *Frieseomelitta varia* were not weighed because body parts are naturally covered in resin. Head width, but not wet weight was used to compare the size of guards and foragers because wet weight might be confounded, e.g., by foragers returning with nectar in their crops.

Worker size variation for all species was measured using the coefficient of variation (CV) for head width corrected for sample size^42^. Worker size variation is routinely used as an overall measurement of worker polymorphism and caste diversity^13,27^. We calculated the CV for each colony and averaged the values to obtain the species specific CV. To estimate queen-worker dimorphism, we used published values where they existed^21^ and measured queen-worker dimorphism where we had access to queens (this was only possible in colonies kept in wooden hives), using the method used by ref. 21: we averaged the length from the tip of the head to the tip of the abdomen of three physogastric queens and three workers per species (1 queen and worker per colony; in *S. bipunctata* only two queens were available).

To obtain a relative and continuous measure of worker differentiation, we expressed the size difference between guards and foragers in relation to overall worker size variation. This DI of a particular species is the ratio between the relative difference in head width between guards and foragers and the overall standard deviation of head width in a colony: $\frac{|{{\rm{HW}}}_{{\rm{Guard}}}-{{\rm{HW}}}_{{\rm{Forager}}}|}{{{\rm{HW}}}_{{\rm{St}}{\rm{Dev}}\,\,{\rm{of}}\,\,{\rm{colony}}}}$. We calculated the DI for head width and head melanization (in *Frieseomelitta*) for each colony and averaged the values to obtain a species specific DI. Thus, a DI = 1 means that the size difference between guards and foragers corresponds to 1 standard deviation of the overall worker sample. The DI provides a straightforward way of comparing the relative degree of differentiation in different species and different morphological traits.

### Quantifying melanization differences in Frieseomelitta varia

We discovered that guards and foragers of three *Frieseomelitta* species were of different color (we also found this in *Frieseomelitta doederleini*; however, we only had individuals of one colony) (Fig. 1f; and Supplementary Fig. 1a). Color differences were most obvious in the head area, the abdomen and the legs. We quantified the differences by measuring the darkness (“melanization”). We photographed each individual in standardized light and magnification conditions and transformed the images to 32-bit grayscale with ImageJ 1.46. We then measured the average gray level in the clypeus. The melanization level *m* was calculated as *m* = 1−$\frac{g}{r}$, where *g* is the average gray level, and *r* is the reference gray level measured on the white background^43^.

### Quantifying cuticle thickness in ***Frieseomelitta varia***

To test whether the darker *Frieseomelitta varia* guards had a thicker cuticle, we caught one guard and one forager from nine different colonies (nine guards, nine foragers) and sectioned the cuticle in three different parts of the clypeal area of the head to measure the thickness of the cuticle using transmission electron microscopy (Jeol-Jem-100cx-II Electron Microscope). Thinly cut head slices were fixed in glutaraldehyde 5% and washed three times in sodium cacodylate 0.1 M. Subsequently, the thickness of the cuticle was measured using ImageJ 1.46.

### Statistical analysis

All tests were done in R 3.0^44^. We used LME models to compare guard and forager head widths in each species. We used a Gaussian error distribution and included colony as a random effect to control for non-independence of data from the same colony^45^. Since we tested 28 species, we corrected *P*-values table-wide (Table 1) to avoid false positives (false discovery rate) using the Benjamini and Hochberg correction^46^. We used an LME and included bee nested within colony as random effects to test if guards in *Frieseomelitta varia* have thicker cuticles than foragers.

To test for allometry within species, we log_10_-transformed all head width measurements and plotted them against the log_10_-transformed cube root of the wet weight. This allowed us to determine whether the relationship between the two traits is isometric or allometric. This is because the slope *b* of the regression log (*y*) = log (*a*) + *b* log (*x*) equals the power term of the geometric relationship *y* = ax^*b*^, with *b* ≠ 1 indicating allometry and *b* = 1 indicating isometry^4,6^. We used reduced major axis regression (model II) to estimate the slopes and to test if they differed significantly from isometry^47^.

When exploring relationships between traits across species, we used GLS models while correcting for phylogenetic dependence and assumed that traits evolve under a Brownian motion model^48^. Continuous variables were logarithmically transformed before analyses^48^. The phylogenetic framework for the comparative analysis of our species (Fig. 1a) relied on the phylogenetic results of Rasmussen and Cameron^18^, complemented with information provided by G.A.R. Melo (pers. communication). The chronogram from Rasmussen and Cameron^18^ was pruned to only include the taxa relevant for this study. Time-proportional estimates for most branch lengths are available^18^, except for the relationships among species of *Frieseomelitta* and *Scaptotrigona*. We used two trees with different sets of branch lengths for species of these genera (divergence time between species = 2 million years vs*.* divergence time between species close to 0) to evaluate the robustness of the results in face of the phylogenetic uncertainty. The two different trees yielded nearly identical results; hence, we present the results of only one of the two trees (divergence time = 2 my).

To study when and how often worker differentiation evolved (i.e., species with larger guards), a Bayesian framework was used for the stochastic reconstructions of character states (with vs*.* without larger guards)^49–51^. We ran 1,000 simulations of a stochastic process of these binary character state changes across the tree branches. A prior probability of equal character state changes was assumed, and the posterior density of stochastically mapped character history was plotted on the phylogeny (Fig. 1). The visualization of the aggregate result of the 1,000 stochastic maps was done using the function densityMap of the R phylogenetics package phytools^52^. Maximum likelihood and parsimony reconstructions were also run, mainly to evaluate the influence of branch lengths on the interpretation of character evolution. Both analyses were run in Mesquite^53^ choosing the option “trace character over trees”: the ML criterion^54^ employed the Mk1 model (all changes equally probable); the parsimony criterion applied Fitch optimization^55^. We used Pagel’s model of the correlated evolution of two binary traits^24^, implemented in the fitPagel function of the phytools package to (1) test if species with negative allometry are more likely to have larger guards and (2) test if known target species of *Lestrimelitta* robber bees are more likely to have guards of increased size. This method controls for the non-independence of closely related species (e.g., among *Tetragonisca* and *Frieseomelitta*). Having larger guards (y/n) was used as the dependent variable, being a target species (y/n) was used as the predictor.

### Data availability

The data supporting the findings of this study will be made available by the authors upon request.

## Electronic supplementary material

Supplementary Information Supplementary Figures and Supplementary Tables

Peer Review File Reviewer reports and authors' response from the peer review of this Article at Nature Communications.
