# Supplementary material for: Repeated evolution of soldier sub-castes suggests parasitism drives social complexity in stingless bees
Source: Nat Commun. 2017 Feb 23;8:4. doi: 10.1038/s41467-016-0012-y (PMC5431902; doi:10.1038/s41467-016-0012-y)
Supplement: Supplementary file 1 — Supplementary Figures and Supplementary Tables [file 41467_2016_12_MOESM1_ESM.pdf]

Supplementary Table 1. Samples sizes, location of collection and information about the natural history of the 28 studied species. No traffic data for both measuring periods was available for *Scaura latitarsis* due to the remoteness of the sampling location. Queen-worker dimorphism (Q-W ratio) is provided for species where information is available.

Species	Nesting type	Brood comb type	Colonies	Bees	Traffic/1min	Q-W ratio	Origin of colonies
Friesella schrottkyi	cavity	combs	8	82	3.3	1.5	Ribeirão Preto - São Paulo
Frieseomelitta flavicornis	cavity	cell cluster	6	76	1.2	2.03	Belém - Pará
Frieseomelitta longipes	cavity	cell cluster	4	28	1.8		Belém - Pará
Frieseomelitta silvestrii	cavity	cell cluster	5	54	9.0		Nova Xavantina - Mato Grosso
Frieseomelitta varia	cavity	cell cluster	6	86	10.6	1.3	Ribeirão Preto - São Paulo
Geotrigona mombuca	ground	combs	2	44	3.8		Ribeirão Preto - São Paulo
Lestrimelitta limao	cavity	combs	2	72	25.0		Ribeirão Preto - São Paulo
Leurotrigona muelleri	cavity	cell cluster	3	60	5.9		Ribeirão Preto - São Paulo
Melipona fasciculata	cavity	combs	6	75	1.8	1.26	Belém - Pará
Melipona flavolineata	cavity	combs	5	61	2.6	1.34	Belém - Pará
Melipona melanoventer	cavity	combs	6	73	1.8		Belém - Pará
Melipona scutellaris	cavity	combs	5	39	1.7	1.3	Ribeirão Preto - São Paulo
Melipona subnitida	cavity	combs	2	48	0.8	1.2	Mossoró - Rio Grando do Norte
Nannotrigona testaceicornis	cavity	combs	4	102	10.1	1.74	Ribeirão Preto - São Paulo
Paratrigona lineata	ground	combs	2	46	7.7		Ribeirão Preto - São Paulo
Partamona helleri	cavity	combs	2	61	22.1		Ribeirão Preto - São Paulo
Plebeia droryana	cavity	combs	5	92	8.5	1.9	Ribeirão Preto - São Paulo
Scaptotrigona bipunctata	cavity	combs	2	94	32.8	2.09	Ribeirão Preto - São Paulo
Scaptotrigona aff. *depilis*	cavity	combs	4	76	31.6	1.5	Ribeirão Preto - São Paulo
Scaptotrigona tubiba	cavity	combs	2	69	8.0		Belém - Pará
Scaura latitarsis	termite nest	cell cluster	3	55			Belém - Pará
Tetragona clavipes	cavity	combs	3	69	91.2		Ribeirão Preto - São Paulo
Tetragonisca angustula	cavity	combs	6	105	27.4	3.3	Ribeirão Preto - São Paulo
Tetragonisca fiebrigi	cavity	combs	6	123	33.6		Santa Helena - Paraná
Trigona fuscipennis	cavity	combs	2	90	269.5		Ribeirão Preto - São Paulo
Trigona hypogea	cavity	combs	2	76	80.0		Ribeirão Preto - São Paulo
Trigona recursa	cavity	combs	3	72	48.6		Ribeirão Preto - São Paulo
Trigonisca nataliae	cavity	cell cluster	2	44	4.5		Nova Xavantina - Mato Grosso

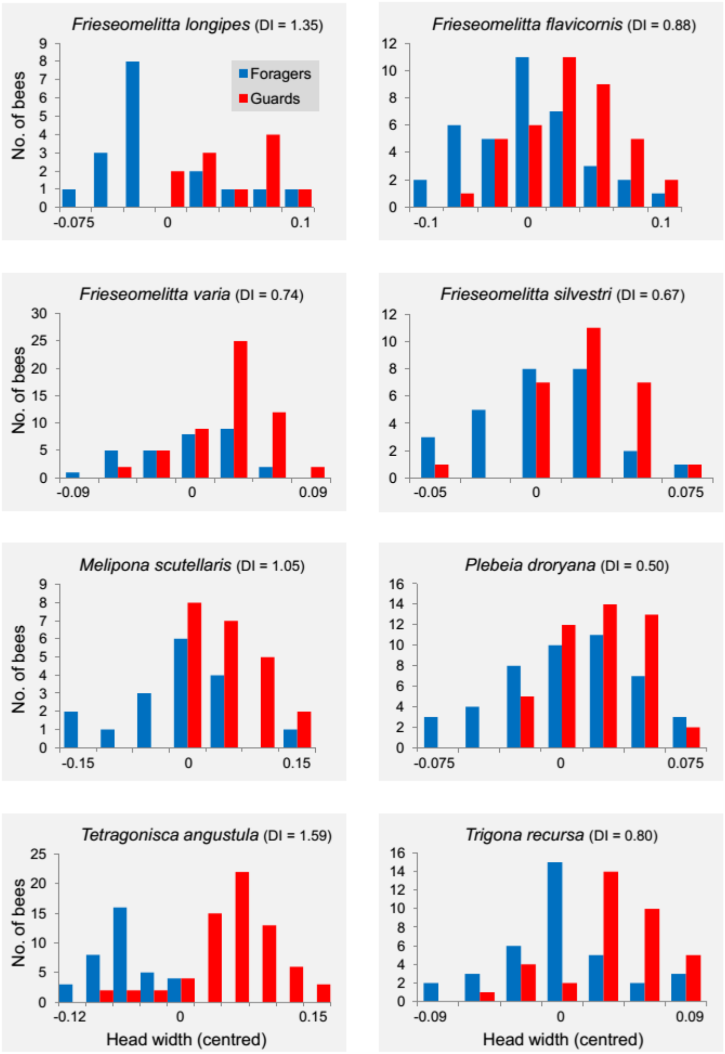

Supplementary Figure 1. Size-frequency distribution (head width) of foragers and guards of eight species with significantly larger guards. Head width data were centred (for each colony separately). Size distribution is bimodal in *Tetragonisca angustula* and *Frieseomelitta longipes,* indicating the existence of two distinct physical sub-castes. The DI (differentiation index) measures the degree of differentiation between guards and foragers in relation to overall worker size variation. For sample size information, please consult Supplementary Table 1.

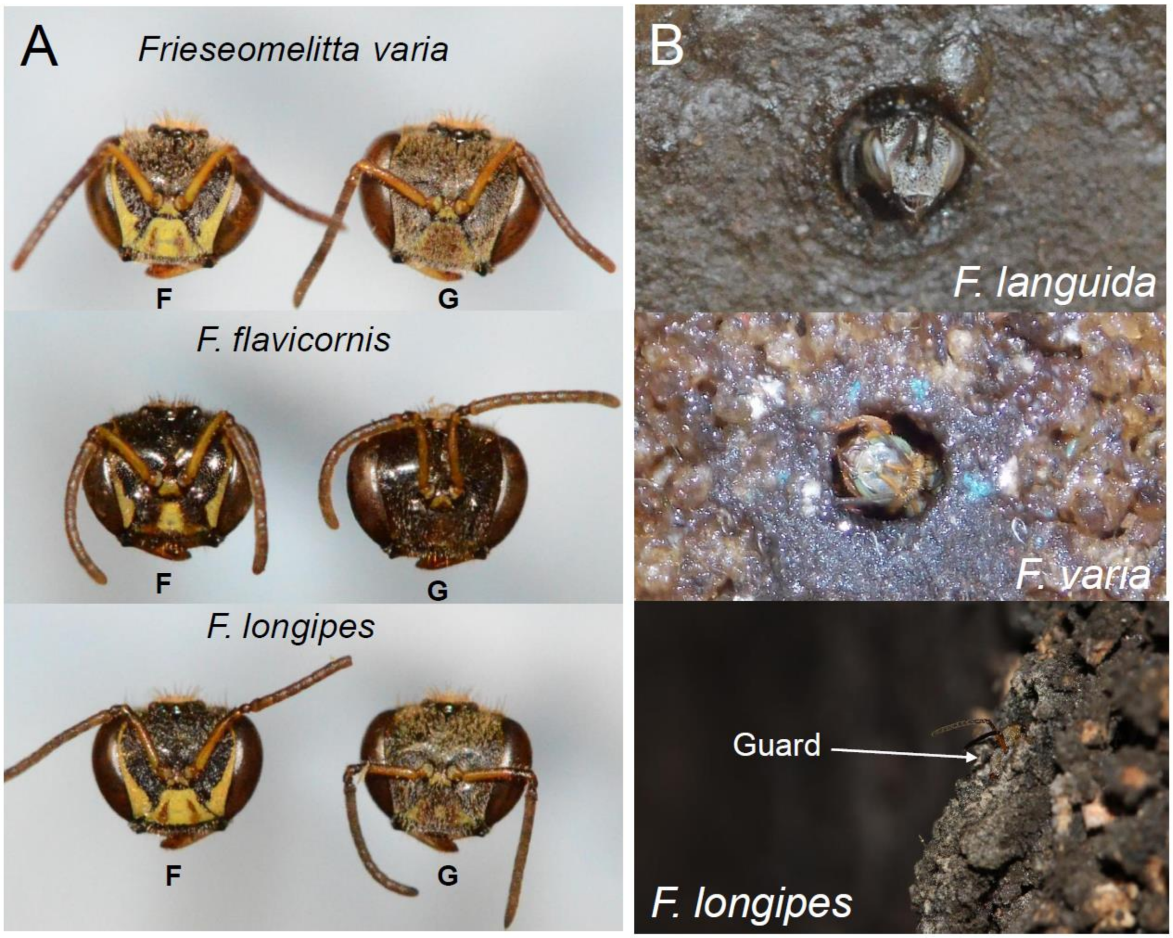

Supplementary Figure 2. (**A**) Head of guards and foragers in three different *Frieseomelitta* species. The differentiation indexes (DI) for melanisation were 1.7 in *Frieseomelitta varia* (Fig. 2c), 1.5 in *F. flavicornis* and 1.85 in *F. longipes*. These were the highest DI's we found (including DI's found for size differences), indicating the existence of two distinct colour morphs (Photos: C. Grüter). (**B**) *Frieseomelitta languida*, *F. varia* and *F. longipes* entrance holes. Entrance holes in *Frieseomelitta* are amongst the smallest in stingless bees. The darker species *F. languida* (and *F. silvestrii*) have darker entrances than species with lighter guards (*F. varia* or *F. longipes*). The workers cover the surface surrounding their nest entrance with sticky substances (Photos: C. Grüter and C. Menezes).
