# Supplementary material for: Repeated evolution of soldier sub-castes suggests parasitism drives social complexity in stingless bees
Source: Nat Commun. 2017 Feb 23;8:4. doi: 10.1038/s41467-016-0012-y (PMC5431902; doi:10.1038/s41467-016-0012-y)
Supplement: Supplementary file 2 — Reviewer reports and authors' response from the peer review of this Article at Nature Communications. [file 41467_2016_12_MOESM2_ESM.pdf]

Editorial Note: This manuscript has been previously reviewed at another journal that is not operating a transparent peer review scheme. This document only contains reviewer comments and rebuttal letters for versions considered at Nature Communications.

REVIEWERS' COMMENTS:

Reviewer #1 (Remarks to the Author):

The major contributions of this study are: (a) to extend evidence for the existence of a morphological defensive subcaste (guards) in workers of stingless bees to a large range of neotropical species; (b) to present a phylogenetic analysis showing that a guard subcaste has evolved multiple times independently in these species, at a time coincident with or later than the origin of the robbing stingless bee genus Lestrimelitta; and (c) to show that species currently targeted by Lestrimelitta are significantly more likely to have larger guards.

These points are novel given that worker subcastes in eusocial insects, while occurring widely in ants and termites, were previously not known from outside these groups, with the exception of a single stingless bee species studied by some of the same authors. Moreover, the study adds to evidence for the ecological determinants of worker subcastes by linking subcaste evolution in stingless bees to a specific selective pressure within the ecology of the focal species, namely the need to have effective colony defence against cleptoparasitic bees. As stated in my review of the previous version of this ms, the findings also have a broader context, given that the evolution of morphological worker subcastes (worker polymorphism) in eusocial insects represents an extreme case of the evolution of division of labour, a feature that manifests itself at several levels of biological organization. In addition, the work appears well conducted, the conclusions are well supported and overall the results should prove influential. In sum, in my view the study does indeed represent an important advance of significance to specialists within the field.

The authors have addressed satisfactorily the comments I made on the previous version. Regarding the present ms, I find it well written but I have some minor suggestions for improving the presentation, as follows:

1. Line 33: Change 'threat of' to 'threat to'.

2. Line 64: Change 'To this aim' to 'To this end'.

3. Line 65: Add a sentence or two to clarify how the 28 study species were selected.

4. Line 78 et seq.: For all cited t values, state the degrees of freedom (applies here and later, e.g. lines 87 and 88). Please check and implement throughout.

5. Lines 80-84: Three species are stated to show a bimodal size distribution, e.g. T. fiebrigi

in Fig. 1C, whereas others, e.g. F. flavicornis in Fig. 1E, are stated to show a unimodal size distribution. I agree that the size distribution illustrated in Fig. 1C looks bimodal and the one of Fig. 1E looks less so, but, given that the distribution in Fig. 1E arguably looks incipiently bimodal, it is not clear whether you have any formal means of classifying the size-frequency distributions as bimodal or unimodal. Please clarify.

6. Line 94: The parenthetical 'yes/no's' in this line read awkwardly and given that you explain the approach in the Methods, could probably be dropped.

7. Line 151: Add comma after 'However'.

8. Line 153: Change 'as foragers' to 'to foragers'.

9. Line 157: Drop the first 'different' (before 'body parts'), so as to avoid repetition, and delete 'to' after 'sizes'.

10. Lines 289-294: You imply that you sectioned the cuticle and then visualised it end-on under the SEM to measure its thickness, but this is not fully clear. Please expand.

11. Figure 1 and Figure 1 legend:
a. In Figs. C, E and G, label the Y axis. One infers it is frequency, but it needs to be explicit.

b. At line 488, the statement that 'values are centred for each colony' seems to apply to Figs. E and G as well as C, so please ensure that this is clear. In addition, even though the values are centred in this way, the X axes of these three panels still need units (SDs?).

c. Add samples sizes (N guards, N foragers, N colonies) for the three distributions (Figs. C, E and G) to the legend.

Reviewer #2 (Remarks to the Author):

I appreciate the comments made and the effort that went into the revision. I am still wondering about the dark faced guards:

"During dusk and dawn, when foraging traffic is low, or when the colony perceives a threat, dark-faced guards can be seen in the entrance."

Could there be two color morphs of guards? I would like to specifically know how the guard function was established. It is curious that a dark-faced individual appear during night. This would suggest another mechanism to me, obviously better camouflaged during darkness than a yellow-faced individual. The only way to establish if the dark-faced are guards is by marking individuals or by observing the activity from the inside through an observation

hive. How was this established without using circular reasoning?

Other than that I still stand with my initial conclusion, that I think it is a well presented study and believe this has a general interest to the readers of the journal.

Best wishes,

Claus Rasmussen

Reply to Referee #1

Referee comment: The authors have addressed satisfactorily the comments I made on the previous version. Regarding the present ms, I find it well written but I have some minor suggestions for improving the presentation, as follows:
1. Line 33: Change 'threat of' to 'threat to'.

Our reply: We have changed this as suggested

Referee comment: Line 64: Change 'To this aim' to 'To this end'.

Our reply: We have changed this as suggested

Referee comment: Line 65: Add a sentence or two to clarify how the 28 study species were selected.

Our reply: Our choices were based on two main factors. First, we tried to select species that are ecologically diverse in order to have a more broadly representative group. Second, we chose more common species as these were easier to find and, therefore, data easier to collect. We added a sentence that further explains our choices (L65-66).

Referee comment: Line 78 et seq.: For all cited t values, state the degrees of freedom (applies here and later, e.g. lines 87 and 88). Please check and implement throughout.

Our reply: We have added this information.

Referee comment: Lines 80-84: Three species are stated to show a bimodal size distribution, e.g. *T. fiebrigi* in Fig. 1C, whereas others, e.g. *F. flavicornis* in Fig. 1E, are stated to show a unimodal size distribution. I agree that the size distribution illustrated in Fig. 1C looks bimodal and the one of Fig. 1E looks less so, but, given that the distribution in Fig. 1E arguably looks incipiently bimodal, it is not clear whether you have any formal means of classifying the size-frequency distributions as bimodal or unimodal. Please clarify.

Our reply: Usually, a bimodal distribution is detected by observing two distinct peaks in the distribution (e.g. Hölldobler & Wilson 2009, The Superorganism; Grüter et al. 2012 *PNAS*). We prefer to be conservative and call the distribution in Fig. 1E unimodal as it is not obvious that there is more than one peak. A Shapiro-Wilcoxon normality test also showed no deviation from normality ($p = 0.53$), which further indicates that the data in Fig. 1E is unimodal.

Referee comment: Line 94: The parenthetical 'yes/no's' in this line read awkwardly and given that you explain the approach in the Methods, could probably be dropped.

Our reply: We removed this as suggested.

Referee comment: Line 151: Add comma after 'However'.

Our reply: Done.

Referee comment: Line 153: Change 'as foragers' to 'to foragers'.

Our reply: Done.

Referee comment: Line 157: Drop the first 'different' (before 'body parts'), so as to avoid repetition, and delete 'to' after 'sizes'.

Our reply: Done.

Referee comment: Lines 289-294: You imply that you sectioned the cuticle and then visualised it end-on under the SEM to measure its thickness, but this is not fully clear. Please expand.

Our reply: Yes, this is what we did. We changed the description and hope it is now clearer (L299-304).

Referee comments: Figure 1 and Figure 1 legend:
a. In Figs. C, E and G, label the Y axis. One infers it is frequency, but it needs to be explicit.

Our reply: We have added this information to the figure.

Referee comments: b. At line 488, the statement that 'values are centred for each colony' seems to apply to Figs. E and G as well as C, so please ensure that this is clear. In addition, even though the values are centred in this way, the X axes of these three panels still need units (SDs?).

Our reply: We now provide this information in the figure legend. We added values to the x-axis and explain what units are used in the figure legend.

Referee comment: c. Add samples sizes (N guards, N foragers, N colonies) for the three distributions (Figs. C, E and G) to the legend.

Our reply: We now provide this information in the figure legend.

Reply to Referee #2

Referee comment: "During dusk and dawn, when foraging traffic is low, or when the colony perceives a threat, dark-faced guards can be seen in the entrance."

Could there be two color morphs of guards? I would like to specifically know how the guard function was established. It is curious that a dark-faced individual appear during night. This would suggest another mechanism to me, obviously better camouflaged during darkness than a yellow-faced individual. The only way to establish if the dark-faced are guards is by marking

individuals or by observing the activity from the inside through an observation hive. How was this established without using circular reasoning?
Other than that I still stand with my initial conclusion, that I think it is a well presented study and believe this has a general interest to the readers of the journal.

Our reply: We agree that the dark-faced guards are a fascinating finding. Our wording was misleading if it created the impression that dark-faced guards are only present at night. The dark faced guards are there during the entire day, we simply wanted to point out that they are more obvious to human observers at times of low foraging traffic, e.g. at dusk or dawn. However, we have captured our guards (the data presented in Fig. 1) at various times of the day, usually between 10 a.m. and 4 p.m. The guarding behaviour in *Frieseomelitta* species is very obvious like in most other stingless bee species (e.g. Roubik 2006, Apidologie; Couvillon et al. 2008, J. Evol. Biol.): guards patrol the area near the nest entrance and often inspect incoming foragers. They will also attack bees from other species if the latter are held close to the entrance and flap their wings if approached with an object like a twig. In many species, guards will also attack humans if they disturb the colony. Thus, the defensive function of the bees at the entrance has been recognized for a long time and their defensive behaviour is easy to observe. Individual marking of guards has revealed that they guard at the entrance for several days (unpublished).
